# From regional climate models to usable information

**DOI:** 10.1007/s10584-024-03693-7

**Published:** 2024-02-29

**Authors:** Julie Jebeile

**Affiliations:** 1 Institute of Philosophy & Oeschger Center for Climate Change Research, University of Bern, Bern, Switzerland; 2 CNRM UMR 3589, Météo-France/CNRS, Centre National de Recherches Météorologiques, Toulouse, France

**Keywords:** Regional Climate Models, Climate change impact, Climate services, Climate uncertainties, Non-epistemic values, Usable or actionable information

## Abstract

Today, a major challenge for climate science is to overcome what is called the “usability gap” between the projections derived fromclimate models and the needs of the end-users. Regional Climate Models (RCMs) are expected to provide usable information concerning a variety of impacts and for a wide range of end-users. It is often assumed that the development of more accurate, more complex RCMs with higher spatial resolution should bring process understanding and better local projections, thus overcoming the usability gap. In this paper, I rather assume that the credibility of climate information should be pursued together with two other criteria of usability, which are salience and legitimacy. Based on the Swiss climate change scenarios, I study the attempts at meeting the needs of end-users and outline the trade-off modellers and users have to face with respect to the cascade of uncertainty. A conclusion of this paper is that the trade-off between salience and credibility sets the conditions under which RCMs can be deemed adequate for the purposes of addressing the needs of end-users and gearing the communication of the projections toward direct use and action.

## Introduction

Today, a major challenge for climate science is to overcome what is called the “usability gap” between the projections derived from climate models and the needs of end-users (e.g. Lemos et al. 2012; Hewitt et al. 2017). Regional Climate Models (RCMs) are expected to bridge the usability gap as they aim to provide local data products and scenario-based projections. RCM-based information is then supposed to address a variety of impacts affecting the environment, infrastructure, agriculture, water management, energy supply, public health, tourism etc., relevant to a range of end-users, encompassing impact researchers, politicians, decision makers, engineers, stakeholders and citizens. Yet what makes climate information usable? To what extent can RCMs provide usable information?

In the climate science community, it is often assumed that the development of more accurate, more complex models with higher spatial resolution should bring an understanding of still ill-known physical processes, as well as projections at fine spatial scales about various kinds of data. It is further believed that such a development of models will  therefore solve the usability gap at some point. However, usable information does not simply emerge from fundamental research in climate science (Jebeile and Roussos 2023); in practice, climate services are developed in parallel with climate science in order to provide usable information. An important reason why the production of usable information does not straightforwardly derive from fundamental climate research is arguable that the improvements of models in terms of accuracy, complexity and resolution are supposed to maximise the credibility of climate information, which is only one dimension of their usability.^1^

In this paper, I assume that, instead, the credibility of climate information should be pursued together with two other criteria of usability, which are salience and legitimacy. A consequence of this assumption is, for example, that highly credible information is of no use for stakeholders if it is not salient or legitimate, i.e. if it fails to meetthe needs of stakeholders or if it is biased toward particular interests. Based on this assumption, I argue that, in the context of the use of RCMs, once one takes seriously all three dimensions of usability, one has to face an unavoidable trade-off between salience and credibility. All in all, the paper is an attempt to offer an epistemological basis for timely science in the service of society, whose priority is to produce usable information rather than merely reliable information.

More precisely, I first put forward a working definition of “usable information” in terms of salience, credibility and legitimacy, and use a number of philosophical distinctions to clarify it in a way that brings out the challenges involved in overcoming the usability gap (Section 2). I then illustrate attempts at overcoming the usability gap by drawing on the Swiss climate change scenarios (Section 3). On this basis, I elaborate on the requirement of salience (Section 4) and illustrate the cascade of uncertainty generated by the model-based production of impact knowledge (Section 5). I then argue that, whereas legitimacy can be maximised without affecting the credibility of climate information, there is a trade-off between salience and credibility that modellers and users have to face. The reason is that meeting the needs of end-users implies going further down into the cascade of uncertainty in that the process generates deeper uncertainties affecting local projections (Section 6). A conclusion of this paper is that the trade-off between salience and credibility provides the assessment conditions under which RCMs can be deemed adequate for the purpose of producing usable information, i.e. of addressing the needs of end-users *and* of gearing the communication of the projections toward direct use and action.

## Usability: salience, credibility and legitimacy

RCM-based information is said to be “usable” in the sense that it is ready to be used in impact studies or to be used in decisions and actions aimed toward mitigation and adaptation.^2^ This section discusses a working definition of usability. The conceptual setting draws upon science and technology studies, as it has been adopted in important contributions to the co-production framework (e.g. Barsugli et al. 2013; Tang and Dessai 2012; Skelton et al. 2017; Kirchhoff et al. 2013; Bremer et al. 2019; Maraun and Widmann 2018).

In order to link scientific information to political decision-making and actions, Cash et al. (2002, 2003) put forward three attributes—salience, credibility and legitimacy—that scientific information must have simultaneously.^3^ Information is salient if it is relevant to the needs of stakeholders and helps them make well-informed decisions. Yet, science may pursue its own internal questions that have no bearing on real-world situations (2002, 4). Information is credible if it meets “standards of scientific plausibility and technical adequacy” (2002, 4) which are epistemic norms widely accepted and used by the scientific community. Information is legitimate if its production is unbiased and fair, and thereby takes into account the values, interests and concerns of different stakeholders (2002, 5).

Information is usable if it is salient enough, credible enough *and* legitimate enough. Yet, the three attributes can either be in tension or complementary with one another. For example, involving decision makers within the production of scientific information may increase salience, as policy-relevant questions are more likely to be raised and answered. This can decrease credibility if science is biased by such an involvement, or conversely, it can increase credibility if it improves the integration of local knowledge in the process through “outside research or assessment” (2002, 6).

Let me now introduce a number of philosophically informed distinctions. From a philosophical perspective, credibility can be said to have an *epistemic* dimension: RCMs are supposed to produce credible outputs which can ground decisions; philosophers would prefer the term “reliability” for “credibility” though. Salience, as I take it, encompasses a *pragmatic* dimension as well as a *cognitive* dimension. On the one hand, information is salient if it meets the needs and the values of end-users. On the other hand, I want to add that information is salient if it is communicated in an intelligible way for the end-users to use the information *directly* without additional cognitive cost. Legitimacy has a clear *ethical* dimension and can be analysed in terms of the values of end-users: the information should not be biased with respect to values that end-users do not share.

These philosophically informed distinctions can in turn highlight the challenges at stake in the resolution of the usability gap, i.e. the “gap between what scientists understand as useful information and what users recognize as usable in their decision-making” (Lemos et al. 2012). In the context of climate services, producing salient, credible and legitimate information would overcome the usability gap. Yet, meeting each of these criteria is a challenge on its own. First of all, meeting the salience requirement requires that the needs of stakeholders be actually elicited and then addressed within the scientific process. Meeting the credibility requirement raises the challenge of the “cascade of uncertainty”, i.e. the accumulation of uncertainties extending from the definition of socio-economic scenarios to the final production of impact knowledge, encompassing the translation of the scenarios into greenhouse gas concentrations, the use of GCMs, the downscaling process in RCMs and the application of impact models (cf. Wilby and Dessai 2010). Meeting the legitimacy requirement involves facing up to the ethical problem that arises when the modellers let their private values influence the production of climate information or when powerful external groups impose their own agenda.

State-of-the-art philosophy of climate science has predominantly focused on the credibility of climate information with a particular emphasis on General Circulation Models (GCMs), the climate models par excellence. Model outputs have been (minimally) meant to be usable provided that they are affected by low and well-quantified uncertainties (in terms of probability or likelihood). Until now, less has been said about Regional Climate Models (RCMs) except that RCMs, being high-resolution non-linear models, are prone to structural model instability (e.g. Frigg et al. 2014; Winsberg and Goodwin 2016; Marina Baldissera Pacchetti 2020). Yet, the extent to which RCMs are (in)capable of delivering usable information should not be evaluated based only on their uncertainties. Philosophers have discussed to a lesser extent the *legitimacy* of climate information. They have shown that the modellers’ own socio-economic and moral values can determine the purposes and priorities of models (e.g. Parker and Winsberg 2018), and that the resulting climate information may differ from the information that would have been produced on the basis of “socially and democratically endorsed values” (to use the expression of Intemann 2015). Philosophers have overlooked the salience dimension; that is why I will elaborate the salience requirement. For that, I will first illustrate an attempt at meeting the needs of end-users based on the Swiss climate change scenarios.

## CH2018: Swiss climate change scenarios

The Swiss climate change scenarios constitute an instructive illustration of how the scientific community interprets “usable information” in a significant effort of communicating climate information to stakeholders (and to the society more broadly). They are typical of national scenarios and services that can be derived from climate science insofar as they make use of RCMs. They are comparable to the scenarios of the UK and the Netherlands, although they slightly differ with respect to “scientific innovation” (e.g. number of used scenarios, choice of models, number of runs, methodologies such as downscaling or quantile mapping, types of output) and “user engagement” in the co-production process (as defined and analysed by Skelton et al. 2017). This case study will remain rather uncritical with regard to how salience, credibility and legitimacy are met. The Swiss approach to communicating uncertain information does not exhaust the possibilities though; they do not include, for example, imprecise probabilities (see e.g. Parker and Risbey 2015), use of likelihood and confidence metrics (Mastrandrea et al. 2010) or narratives (Dessai et al. 2018).

### Presentation

The Swiss scenarios are particularly challenging. On the one hand, the near-surface air temperature has increased by about 2.0 °C in Switzerland between 1864 and 2017, while it has increased by about 0.9 °C globally (CH2018 2018, 7). On the other hand, the complex topography, notably due to the Alps, creates “a diverse climate with large elevation gradients, spatial heterogeneity, and small-scale phenomena” (CH2018 2018, 6).

In this context, local projections about the new physical conditions, in terms of temperature and precipitation for example, and about future seasonal trends (e.g. warmer and drier summers, rainier and less snowy winters) crucially serve as inputs for the study of impacts on species (including human beings), ecosystems, environment, agriculture or tourism, with which the physical climate is connected. In turn, these impacts may have feedback effects on the physical climate. To give just one example, with heat stress and more frequent droughts, soils will likely become less wet, thus potentially transforming forests from carbon sinks to carbon sources (OcCC and ProClim 2007, 158).

In 2011, a first set of climate change scenarios was delivered (OcCC and ProClim 2007; CH2011 2011; CH2014-Impacts 2014), and in 2018, an improved, broader and more user-oriented set has been produced (CH2018 2018), on which this paper focuses. The 2018 Swiss scenarios delimit five model regions covering the territory at three relevant time periods (i.e. 1981–2010, 2011–2069 and 2070–2099). They follow three typical emissions scenarios, the Representative Concentration Pathways (RCP), to which the level of radiative forcing reached by the end of the century is assigned. RCP2.6 is an ambitious mitigation scenario that is compliant with the 2 °C mitigation Paris Agreement. RCP8.5 is an unabated emissions scenario and constitutes in this regard the worst-case scenario. RCP4.5 is an intermediate scenario.

Within the model chain, recent EURO-CORDEX simulations, which are RCM simulations of the European region, are taken as a starting point; they themselves have been produced using GCM projections as drivers (Jacob et al. 2014) with the purpose of providing relevant information (as a CORDEX project, see Giorgi et al. 2009). First, a selection of the EURO-CORDEX simulations is made: those that present “problematic or unrealistic” results for Switzerland are removed (CH2018 2018, 49–53). Second, the spatial resolution of EURO-CORDEX simulations is 12 km and thereby too coarse for local impact assessments. Therefore, the Swiss scenarios aim to downscale these simulations to a spatial scale relevant for end-users (i.e. to a 2 km × 2 km grid), and at the same time to correct for the possible misrepresentations of the Swiss climate due to topographical specificities. This method uses quantile mapping that corrects for the biases conveyed by the GCMs at the model grid scale: it assumes “a constant relationship between spatial averages on the model grid scale and local values” and infers a “transfer function”, and this function aligns the simulated variables with the observations (CH2018 2018, 23).

CH2018 datasets, available online (NCCS 2023), finally provide meteorological *variables* (e.g. temperature, precipitation, relative humidity and near-surface wind speed) at daily, seasonal and annual resolutions; climate impact-relevant *indices* (e.g. temperature indices such as hot days, tropical nights, frost and ice days); and frequency of climate *extremes* (e.g. temperature and precipitation extremes). While indices refer to events likely to happen, extremes are rare events that have particularly harmful human and environmental consequences. They are obtained with similar methodologies (CH2018 2018, 104); in particular, extremes correspond to the tails of the statistical distribution of the variables of interest, from a defined threshold (CH2018 2018, 105).

**Fig. 1:**
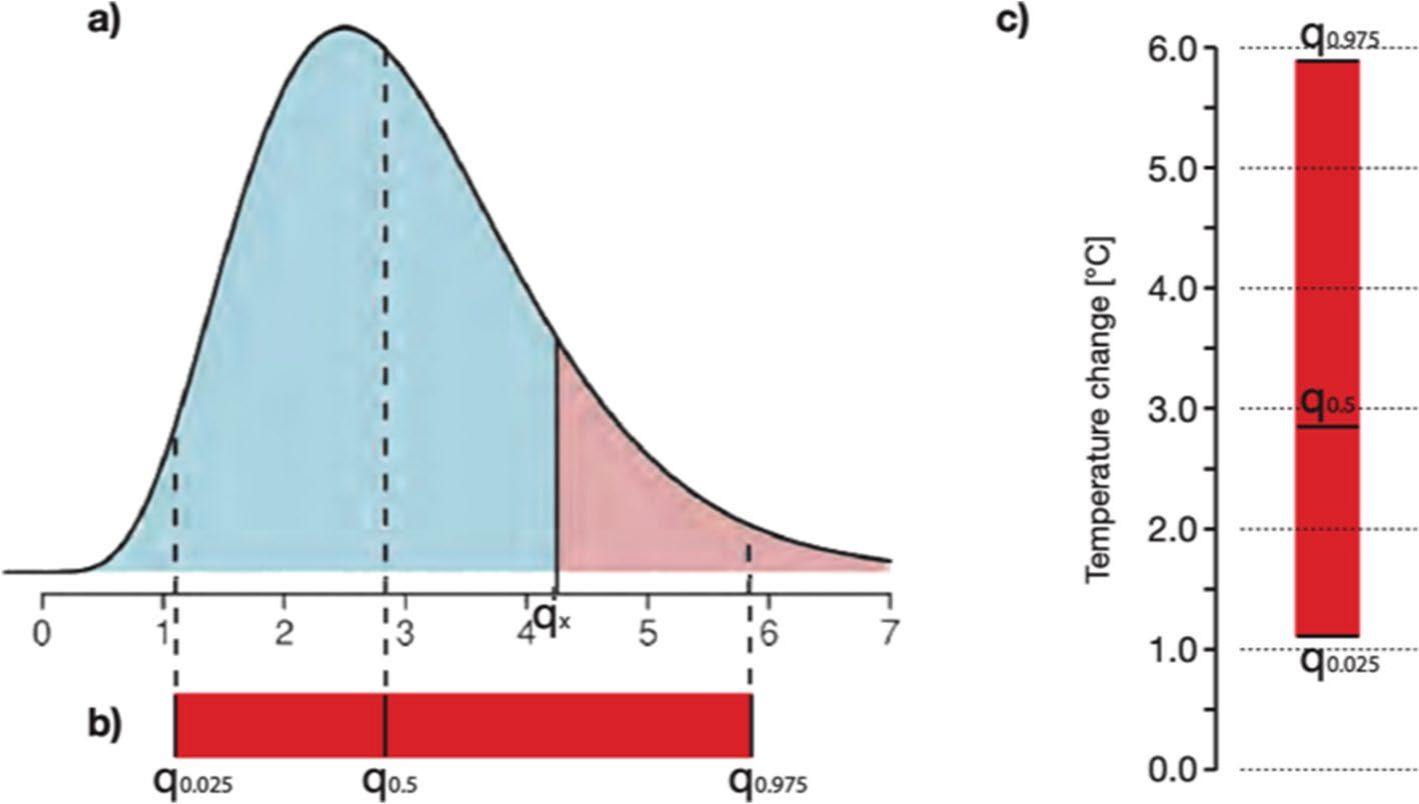
Probability distribution of temperature characterised by the median (*q*_0.5_) and the 95% confidence interval (*q*_0.025_ to *q*_0.975_) (OcCC and ProClim 2007, 14, Fig. 2)

The outputs are associated with uncertainty estimates that are quantified by an ensemble-based approach, as is commonly the case in GCMs. It is assumed that the spread of the joint simulations for a scenario, called model spread, quantifies, albeit imperfectly, the model uncertainty. From the resulting model spread, for each variable of interest and climate statistics, multi-model medians and probability density functions can be calculated as illustrated in Fig. 1.

But that is not yet the whole story. At this stage, usable information is not fully obtained. Multi-model medians and associated probabilities for the simulated variables are just a part of the expected output. More has to be done to meet the needs of end-users. Pre-selected outputs are calculated and edited for this very purpose, while others are inferred from RCMs, and, as we will now see, they are in practice expressed in a specific way that deserves attention. As I take it, the way outputs are expressed reflects how scientists endeavour to satisfy the urgent need for salient data while dealing with uncertainties in RCM projections.

### Need for relevant data

The choice of the relevant variables, indices and extremes depends of course on the specific climate of the geographical location of the region being investigated. They meet the needs of end-users when they address the climate-related risks that might occur in the region, but also the variables of direct interest for the impact studies in this region. In Switzerland, because of the mountainous landscape, they importantly include the volume of alpine glaciers, the zero-degree line in winter, the number of snow and snowfall days and the frequency of heavy rainfall extremes.

Indices and extremes of interest are then used to assess the future climate-related risks. For instance, in winter, the higher snow line will shift snow to rain, increasing rainfall and thereby the risk of flooding (CH2018 2018, 138). In summer, the more frequent and longer heat waves will lengthen the vegetation period and the evaporation demand, thus making soils drier and intensifying agricultural droughts. Day and night heat stress, further amplified by urban heat island effects, increases body temperature and can cause heat-related illnesses.

That said, the RCM outputs sometimes need to be converted into other relevant data for impact studies. One example is drought. There is no unique definition of drought, but rather several definitions—whether meteorological, agricultural or hydrological—which lead to a plurality of indices (CH2018 2018, 132). Meteorological drought traditionally refers to a deficit of precipitation, agricultural drought to a deficit of soil moisture and hydrological drought to negative anomalies in streamflow, lake and/or groundwater levels. They can more or less easily be derived from the CH2018 projections; precipitation deficit gives meteorological drought, which in turn, with evapotranspiration, can lead to agricultural drought, which in turn, with pre-event soil moisture, surface water or groundwater storage, can lead to hydrological drought.

Another example of conversion in CH2018 is the conversion of near-surface air temperature into wet bulb temperature. Wet bulb temperature can directly document the extent to which human beings physically suffer from heat waves, the limit of survivability for healthy humans being set at 35 °C. High external temperature can generate heat stress in the human body: high external temperature leads the body to generate sweat evaporation in order to maintain a healthy internal temperature, but high external air humidity limits sweat evaporation, and makes the internal temperature increase (CH2018 2018, 31).

**Fig. 2:**
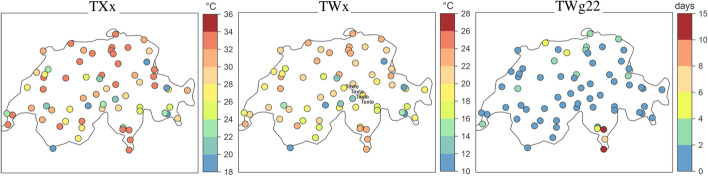
Mean annual maximum temperature (TXx), wet bulb temperature (TWx), and number of days with TW above 22 °C (TWg22) in summer for 1981–2010 observed at 67 Swiss stations (CH2018 2018, 31, Fig. 3.6)

Because the risk to humans depends on temperature and humidity, wet bulb temperature derives from temperature and humidity by means of thermodynamic equations or empirical formulas (CH2018 2018, 31). Figure 2 presents a Swiss map with the mean annual maximum temperature (TXx), the wet bulb temperature (TWx) and the number of days with the mean annual maximum temperature above 22 °C (TWg22). This figure thus provides immediate information about where human beings will suffer the most from heat stresses.

*Thresholds* are often assigned to the datasets. They are obviously subjective choices and are therefore value-laden in that they depend on our perception and acceptance of risks. One example we have just encountered above is the biological limit of survivability for healthy humans set at a wet bulb temperature of 35 °C. Of course, such a biological limit must be avoided as it corresponds to intolerable harm. The number of summer days above 22 °C is used to quantify intense heat stress (CH2018 2018, 31); heat stress is the human body’s failure in maintaining its internal temperature constant due to high external temperature and humidity. In Fig. 2, it appears that this threshold is exceeded in a few locations, and particularly in the low-lying stations in Ticino. The specification of thresholds here seems similar to the specification of thresholds of radioactive toxicity in the safety of basic nuclear installations, which is based both on considerations about biological damage for living beings and on the level of risk that society is ready to endure. Other thresholds are defined in the CH2018 report: for example, frost days and hot days are assigned thresholds of *T* < 0 °C and *T* ≥ 30 °C respectively (CH2018 2018, 38); the frequency of warm days is calculated for days exceeding 25 °C; 1 mm day^−1^ is the minimal precipitation beyond which daily events are “wet” and below which daily events are “dry” (CH2018 2018, 107).

As I will highlight, meeting the needs of end-users in terms of relevant data comes with additional uncertainties. First, though, let us look at how the communication of data is in practice made intelligible, as the way data are expressed, while dealing with their uncertainties, is crucial for properly informing decision-making.

### Communication of data with uncertainties

The establishment of usable information requires a choice of relevant data, and this choice can be made during stakeholder dialog; this dialog enables collaborations between providers and users of the RCM products for them to define collectively the choice of methods and datasets. A further requirement is the way the associated uncertainties are quantified and communicated. As presented in Section 3.1, uncertainties associated with RCM projections are often expressed in terms of model medians and uncertainty ranges. Data are calculated at daily, seasonal and annual resolutions. In the CH2018 report, decision-relevant ways of expressing information are also used to manage uncertainties in projections. These ways include (i) highlighting the minimal and strongest changes by exploring the socio-economic scenarios, (ii) quantifying the intensification of warming effects and (iii) providing analogues of future climates in Swiss cities based on the present climates of other cities in Europe.

**Fig. 3:**
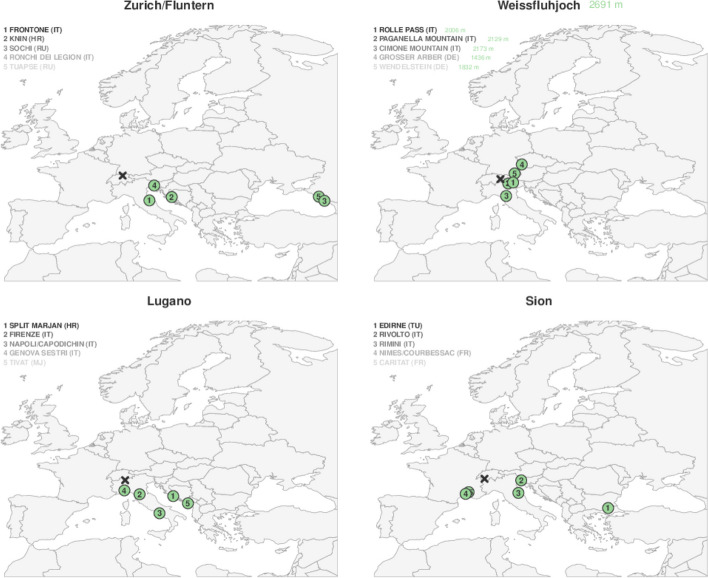
Climate analogues for the end of the century (2085) and under RCP8.5 (CH2018 2018, 167, Fig. 10.1)

(i) The different scenarios convey information about the various possible strengths of the warming. On the national scale, we learn that “The strength of the warming at the end of the century (2070–2099) relative to the reference period (1981–2010) highly depends on the emission scenario considered, with a summer increase of 0.7–2.4 °C for the mitigation scenario RCP2.6 compared to 4.1–7.2 °C for the unabated emissions scenario RCP8.5. Thus, the warming from now until the end of the century will be about three times larger without mitigation” (CH2018 2018, 8). For an impact assessment study, it is therefore important to properly select the appropriate scenario on the basis of which large-scale climate phenomena cause the impact being investigated (CH2018 2018, 181).
(ii) Common quantifiers are also assigned to variables, indices and extremes. The quantifiers include frequency, intensity and duration period in days; these are common statistics derived from the projections. An example of frequency indices is the number of hot days, and an example of intensity indices is the intensity of a precipitation event that, on average, occurs once every 10 years.
(iii) The RCM outputs can also be used to find the so-called climate analogues: the idea is that the future climate could be similar to the present-day climate somewhere else on the planet. For example, the analogues for the future Swiss climate can be found in present-day climates near the northern Mediterranean coastline (CH2018 2018, 11 and 166–168). Figure 3 presents four sites in Switzerland (marked by a cross) which are Zurich/Fluntern, Weissfluhjoch, Lugano, and Sion, and, for each site, five possible climate analogues (assigned with numbers encircled by a green disk) whose names are listed in the upper left part in decreasing order of similarity.

In OcCC and ProClim (2007), this type of analogical reasoning, based on the comparison of temperatures and precipitation, constitutes a powerful way of making users imagine how the future climate of some cities will likely be. Thus, we can read that “there are stations in Switzerland and abroad where today’s temperature conditions match those that specific locations will experience in 2050 as a result of the warming. In a weak warming scenario, the temperatures in Zurich will resemble those of today’s conditions in Sion, with medium warming they will be similar to today’s conditions in Magadino, and with strong warming they will be similar to today’s conditions in Torino” (OcCC and ProClim 2007, 16). This type of reasoning is to some extent similar to the inductive and rather genealogical methods used in astrophysics where, based on the current classification of observed objects (galaxies, stars etc.), scientists infer the past states that newly discovered objects have likely gone through, or the future states that they will likely go through, depending on their age. Astrophysicists thus use the statistical distribution of objects into categories corresponding to different stages of evolution (of a star, for example, from its birth to its disappearance) in order to reconstruct the times spent in each phase of the evolution—because, of course, we cannot follow the course of the evolution of a star over time given the long time scales involved.

## Requirements for salient climate information

I have highlighted what the relevant climate data are for Switzerland and how they are communicated with their uncertainties based on the 2018 technical report of the Swiss scenarios. This way, I have illustrated how salience is met within the report. On this basis, I will now put forward four pragmatic requirements that salient information must meet.

Information is salient if it meets the needs of end-users. Needs are value-driven: they depend on the interests, expectations and priorities of the end-users. Values can be social or ethical considerations; they can appear as an expression of political intrusion (in the form of preferences or interests) within the scientific sphere (which is usually conceived as a place for the production of objective knowledge). Examples of values include the protection of biodiversity, the environment, public health or economic growth. We may prioritise them as a function of what we value the most. In turn, values may determine the types of data that are the most relevant to us. Because needs are value-driven, it is hard to define the needs of end-users in advance. What matters for end-users and stakeholders may partly depend on circumstances that are difficult to foresee; the expression of their needs may arrive at the stage of co-production of climate information or even later. In addition to that, user groups can change over time. Nonetheless, I assume that some requirements for the creation of usable climate information may be seen as merely pragmatic (albeit being value-driven).

### Local

A large quantum of usable information must be local, i.e. relevant at the scale of a territorial unit covered by a local policy and administration: this can be a city, a metropolis, a canton or a municipality within a country (see Oreskes et al. 2010). The scale in question must be pertinent for political decision-making with targeted local actions on the territories.

In this context, RCMs seem adapted to provide usable information for adaptation as they aim to produce local projections.^4^ That said, GCMs can also help in reaching this aim. On the one hand, GCMs yield averages about the physical state of the climate at the scale of the planet. Global projections can constitute actionable information on their own, but mostly for mitigation, rather than adaptation, and at an international scale. Indeed, they feed the IPCC Assessment Reports, assist in the development of global climate-related policies and serve in the negotiations at the UN Climate Conference. Global projections inform us about the state of common goods (e.g. our atmosphere, our air quality). Thereby, they provide indicators on how much collective effort is required at a global scale to maintain acceptable living conditions overall, and how much effort must be distributed to alleviate international injustices caused by the impacts of unequal greenhouse gas emissions, in particular between the Global North and the Global South. On the other hand, GCMs can contribute to the production of local projections in that ensembles of GCMs can actually be helpful to distinguish the climate change signal from internal variability affecting local projections. Indeed, despite higher representational accuracy in models, climate noise is exacerbated at local scales and makes the production of local information particularly difficult. In order to remedy this difficulty, ensembles of GCMs are for example used in the case of local projections of precipitation in Western Europe, including the Alps (Aalbers et al. 2018). Finally, some users find it sufficient to downscale GCMs with a quantile mapping method in order to obtain the local information they find useful, and it is also considered that RCMs are not always the best candidates for providing local information as they sometimes fail to quantify uncertainties, such as rare extremes (see Lenderink et al. 2023).^5^

### Complete

Local information should also be complete; i.e. it should cover the different relevant territorial units for a national policy of adaptation. If the data are local but too scarce, they certainly will not be sufficient to prepare adaptation strategies within the whole country. In Switzerland, there are currently 58 observatory stations for which projections are expected. But, beyond this, the needed set of projections must cover the territory in accordance with its administrative division into territorial units of political action. Let this administrative division be a division into cities, municipalities, cantons and other federations. Each territorial unit—municipalities and cantons in the case of Switzerland—should have equal access to information. This requirement can be met by an adequate choice of grid in the RCMs, for which each territorial unit is at least covered by the nodes of the grid.

As I take it, the first two requirements for usable information—being local and complete—are pragmatic. They are oriented by a territorial division driven by the mapping defined by the executive within a country. That said, they are not absolutely neutral regarding the interests of the country. At the international scale, there is a risk of “epistemic inequality” that has been initially described by Parker and Winsberg (2018), Jebeile (2020) and Jebeile and Crucifix (2021): value influence may shape the purposes and priorities in GCMs, and consequently, models may provide better projections for the geographical regions and corresponding variables that are highly valued by the modellers. This is an ethical problem in that the less informed populations may well be the most vulnerable to climate change. The epistemic inequality may also apply at a national scale since a division of a country into territorial units is political. Even in a country which is not in a state of civil war or ethnic conflict, this may raise issues of fairness in how well countryside and urban areas are informed for example, and a region might require further differentiation into sub-regions for democratic and ethical reasons.

### Relevant

Information must be relevant in that it must meet the need for relevant physical data (at a local scale for adaptation and at a global scale for global mitigation). There are data which logically derive from the fundamental equations of physics on which climate modelling is based. They are derived, for example, from the equations of thermodynamics (e.g. radiative force, temperature), the equations of fluid dynamics (e.g. wind speed, pressure) etc. However, they may not directly interest end-users. Hence, it is important that, at the stakeholder dialog, the specific end-users’ needs for physical data are expressed and taken into account. The relevant physical data can then be edited or calculated. They can serve as inputs in impact models, or directly for decisions and actions directed toward adaptation. In Section 3.2, I have provided illustrations of typical needs in terms of climate variables in the case of the Swiss scenarios.

### Intelligible

Information is usable if its communication to end-users is geared toward political action in the context of adaptation to climate change. The information should be presented in a form that is *directly* understandable by a broad set of users. The communication of this information, along with the relevant data, expresses the associated uncertainties. The intelligibility of claims about uncertainty might require transparency regarding the idealisations underlying the methods used to produce the climate data.^6^

Thus, in particular, the communication of the uncertainties must be intelligible. Generally, climate scientists are asked to quantify uncertainties and estimate probabilities; however, as we have seen in Section 3.3, there are in practice other ways of expressing the data with their associated uncertainties. Briefly, they include (i) the set of possible numerical values for the same datum under different socio-economic scenarios; (ii) the frequency, intensity and duration of indices and extremes; and (iii) climate analogues that provide an exemplification of the future climate of a city through its comparison with the present climate of another city somewhere in the world.

To sum up, salient impact knowledge should generally be (1) *local* (i.e. about future local changes), (2) *complete* (i.e. covering the region of interest), (3) *relevant* (i.e. about variables of direct interest for end-users) and (4) *intelligible* (i.e. easy to understand while being associated with uncertainties).

## Cascade of uncertainty

My goal now is to show that producing salient information from RCMs comes with additional uncertainties compared to uncertainties affecting GCM projections. In other words, when modellers seek to meet the needs of end-users, they create new uncertainties (although only to some extent, because some bias correction is also incorporated later in the model chain). These new uncertainties are thus typical of climate science transitioning to a science *in the service of* society.

**Fig. 4:**
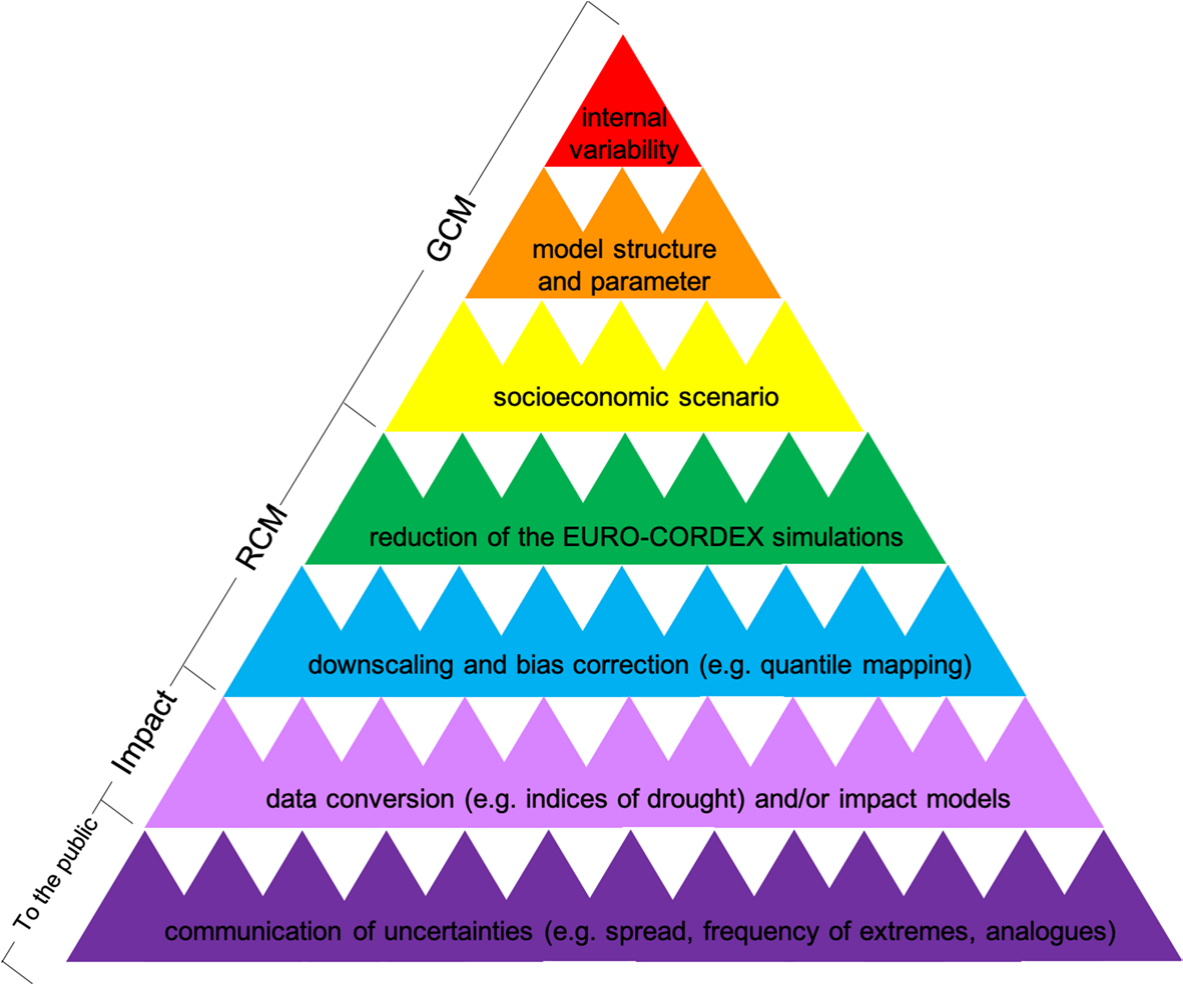
Adaptation of the “cascade of uncertainty” by Wilby and Dessai 2010 (Fig. 1.A, 181)

In order to see this, it is important to have in mind the different steps in the process of producing usable information, i.e. the model chain: this goes from GCMs, to CORDEX simulations, to RCMs by downscaling techniques and bias corrections, to possible additional data conversion and/or to impact models. Figure 4 illustrates this cascade of uncertainty for the Swiss scenarios; it is an adaptation of the cascade of uncertainty originally drawn by Wilby and Dessai 2010 (Fig. 1.A, 181).

Uncertainties in GCMs include uncertainty arising from internal variability, which is due to the spontaneously varying nature of the climate system; model uncertainty, due to models being incomplete, idealised and parameterised; and scenario uncertainty, due to the uncertainty of future decisions as concerns social and economic development. The uncertainty arising from internal variability can be described as ontic uncertainty as it is due to “the climate system’s having some irreducibly statistical or indeterministic character” (Parker 2013, 216). The others can be described as epistemic uncertainty as they concern our state of knowledge.

In RCMs, there are additional uncertainties because of the initial reduction of the set of the EURO-CORDEX simulations. Such a reduction has, for example, “a large effect on the spread in temperature and precipitation projections across the Alpine region” (CH2018 2018, 66). Other uncertainties are due to the downscaling process and the bias correction applied to generate local projections (see Jebeile et al. 2020 for further details). In other words, RCMs might present a higher level of uncertainty than GCMs insofar as RCMs are derived in part, via downscaling techniques, from GCMs (see CH2018 2018, 66 for a summary of the uncertainties implied by the series of processing steps in the CH2028 scenarios). As said in Section 4, a major difficulty with RCMs is notably to distinguish the “noise” produced by natural variability at local scales from the genuine signal of climate change. Thus, it remains difficult to attribute phenomena whose representations are usually affected by the limited level of resolution and the lack of process understanding (such as low stratus, storms, hail, thunderstorms, tornadoes or pollen migration) to climate change.

A word of caution is also given by the authors of the CH2018 report regarding the limitations of quantile mapping for applications of climate impact research since quantile mapping “is a purely statistical and data-driven method” that “does not incorporate physically based knowledge” (CH2018 2018, 102).

It should also follow from the description provided in Section 3.2 that the conversion of data—such as indices of meteorological, agricultural or hydrological drought—may generate residual errors in that they are based on different approximate physical formulas. Within the model chain, subsequent climate impact models based on RCM scenarios also come with their own kinds of uncertainties.

Furthermore, as described in Section 3.3, uncertainties are communicated in terms of (i) range of scenarios; (ii) frequency, intensity and duration in days, of variables, indices or extremes; and (iii) climate analogues. The ways of communicating uncertainties raise specific challenges. First of all, the range of scenarios leads to a set of projections whose spread is used to quantify uncertainties. Because the EURO-CORDEX models are to some extent interrelated and do not comprise all the theoretically plausible model formulations,^7^ the resulting model spread is not representative of the overall uncertainty. Therefore, an additional percentage of uncertainty (here 5%), estimated by expert judgment elicitation, is applied to the model spread, as is often the case in GCMs (e.g. Thompson et al. 2016). Before this, given the initial selection of EURO-CORDEX simulations, missing simulations are substituted by pattern scaling, a time-shift-based method, in order to maintain the statistical significance of the initial set of simulations (CH2018 2018, 51).

Second, specific errors are generated by the use of climate analogues. As has been remarked, “these results provide an illustrative but only approximate picture of future climate change in Switzerland” (CH2018 2018, 168). The main reason for this is that the analogy relies on a comparison between seasonal mean values of temperature and precipitation only, while other variables like radiation and insolation, depending on latitude, may be also important in the identification of analogues (CH2018 2018, 168). Furthermore, only a sample of cities is considered (2471 sites in Europe in the report) while other cities in the world could be better analogues of Swiss cities.

In a nutshell, meeting the needs of end-users generates additional uncertainties in that these needs concern local and complete projections, as well as relevant data, and should be communicated in an intelligible way. This leads to a trade-off, as I will now argue.

## Reconciling salience, credibility and legitimacy

As said, a common assumption underlying research programmes in climate science is that more accurate, more complex and higher resolution models should provide more reliable projections and thereby are more likely to offer usable information. Thus, parameterisations are replaced by theoretical equations in order to improve accuracy. More climate processes are included in models, thus increasing model complexity. Spatial resolution is made finer to get local projections. It is expected that the resulting projections will support local decisions and actions, and serve a broad range of purposes. However, usability is a matter of degree and depends on three dimensions, i.e. salience, credibility and legitimacy. In order to produce usable climate information, can one simply maximise all three dimensions?

What makes information legitimate depends on how well the values of the users are elicited in practice and taken into consideration within the models. Legitimacy can therefore be increased, if, for example, the group of users participating in the stakeholder dialog is sufficiently socially diverse and representative of multiple alternative viewpoints. That way, the interests of manufacturers and politicians having powerful voices are not heard over the positions of usually underrepresented populations. Legitimacy can be further increased if the elicitation of values is executed via a democratic and fair process, through open deliberations for instance, so that the elicited values are socially and democratically endorsed (Intemann 2015; Lusk 2020). Parker and Lusk (2019) provide an account of how to produce usable climate information on the basis of the values of users and, at the same time, to enable the users to avoid particularly undesirable outcomes. In doing so, they illustrate a way in which legitimacy can be maximised although projections are uncertain. As they contend, “climate services should consider not just what users want to know, but also which errors users particularly want to avoid” (2019, 1643). The management of the risk of errors should concretely consist of implementing a strategy along the lines of the inductive risk argument for which values can be used to choose between methodologies which are known to err in different ways (Douglas 2000, 2009). In the context of climate services, the values of end-users should be expressed on the occasion of collaborative consultations between modellers and users, and then used to choose, among several methodologies, the ones which are less likely to err in ways that would have particularly bad consequences from the users’ perspective.

However, salience can be maximised only at the sacrifice of credibility.^8^ As explicated, salient information in the context of climate services should be local, complete, relevant and intelligible to a wide audience. Yet, if the top-down approach—from RCPs to GCMs, to RCMs—is adopted in order to reach locality and completeness, one has to face the cascade of uncertainty. Furthermore, the search for relevance and intelligibility also entails a loss of credibility. The production of practical and low-cognitive-cost information, i.e. information that one can easily grasp without making additional inferences, generates additional uncertainties. This is for example the case for drought indexes, wet bulb temperature and climate analogues.

The above-mentioned model improvements are supposed to reduce some sources of uncertainty in the cascade: for instance, more accuracy aims to decrease structural and parametric uncertainty; higher resolution aims to avoid uncertainty due to downscaling and bias correction. But some other sources of uncertainty, due to internal variability, the choice of socio-economic scenario, the design of the ensemble, the process of data conversion and impact modelling and the communication of uncertainties, are roughly unchanged. The cascade of uncertainty does not imply that local projections are not credible per se, but that they are certainly less credible than global projections as their production entails additional uncertainties. In this context, more uncertainties mean lower credibility for *local* information. The cascade of uncertainty is not a cascade of newly explored uncertainties, but occurs along the “horizontal” model chain that goes from global to local scales.^9^

Reaching locality, completeness, relevance and intelligibility involves RCMs, statistical techniques of downscaling and data conversion, and all of these modelling procedures come with extra uncertainties. In this sense, there is a trade-off to make between salience and credibility. Importantly, this trade-off may not hold for alternative bottom-up approaches. Such approaches for climate adaptation policy do not aim to provide probabilities, and are not based on climate models. This is the case of the vulnerability approach that starts from social or biophysical vulnerabilities; explicates the mechanisms behind the past and present conditions that created those vulnerabilities; and, this way, informs about future risks (see Dessai and Hulme 2004). The possibilist reasoning (e.g. Katzav 2014) and the storyline approach (e.g. Shepherd 2019) are also alternative solutions to probabilities (see Katzav et al. 2021).

The trade-off between credibility and salience in the top-down approach certainly constrains the degree of usability of climate information. As imprecise as information can be, its virtue may still rely on the purposes of meeting salience and legitimacy. A striking example is the case of climate analogues. Even though climate analogues are produced at the cost of an exacerbated cascade of uncertainty, they provide salient information, and enable one to directly figure out the local consequences of climate change, as well as some possible adaptation strategies. Such information is not communicated in terms of probabilities or other measures of likelihood. Rather, it is conditionalised on the likelihood of the scenario on which it derives. Such a trade-off between salience and credibility is certainly justified by the context of an emergency. We have at our disposal local information, which, despite uncertainties, can ground our decisions and actions regarding climate change adaptation. This should encourage us, the users, to adopt a specific epistemic attitude toward the information when assessing the uncertainty, whether this attitude is absolute confidence, relative confidence, reasonable doubt etc. It is not necessarily measured quantitatively, but lies partly in the appraisal of the users. Thus, for example, we understand a prediction in a different way than we understand a projection, which, as we know, is conditioned by the hypothesis of a possibly misleading scenario. While it is supposed that one can assign probabilities to predictions, projections carry in themselves a conditional statement, in the sense that, unlike predictions, projections are conditioned by a given scenario. As the IPCC claims, “The goal of working with scenarios is not to predict the future but to better understand uncertainties and *alternative futures*, in order to consider how robust different decisions or options may be under a wide range of possible futures” (IPCC 2019, emphasis mine).

## Conclusion

The purpose of this article was to elaborate on the salience dimension as regards usable climate information. I have shown that, in order to produce salient information, it is necessary to take into account the needs of the users, which in itself generates a number of uncertainties. This is not to say that this information is useless or that it cannot be used for decision-making, but the way in which uncertainties are communicated—in terms of scenarios, or frequency, intensity and duration of variables, extremes and indices, or climate analogues—appears to be absolutely complementary to the calculation of the probabilities. While legitimacy and uncertainties can be reconciled, communicating usable information requires making a trade-off between the salience and credibility of the information, and this trade-off calls for optimisation. The resolution of such a trade-off is key to producing usable RCM outputs that address the needs of end-users and make direct use and action possible in a timely matter.

## Data Availability

Not applicable.

## Footnotes

1 In practice, such a development of models faces limited available computer power and process understanding. Furthermore, as pointed out by a reviewer, higher resolution does not always come with more reliable information; sometimes it does, e.g. in the study of small-scale showers, sometimes it does not, e.g. in the study of winter storms.

2 The expression “actionable information” seems limited to the context of decisions and actions only.

3 There are other definitions, though. For example, Hewitson et al. (2014) propose that usability subsumes plausibility, credibility, and defeasibility. In Asrar, Hurrell and Busalacchi (2013, 10), usable information is deemed to be “timely, accessible, and easy to understand”. Giorgi (2020) adopts the “3R approach” in which actionable information meets the standards of robustness, reliability and relevance.

4 CORDEX simulations are said to provide projections on “regions”, but the latter term here refers to rather continental-like territories.

5 I thank a reviewer who pointed out this aspect.

6 Transparency is an important requirement for quality, as highlighted by Baldissera Pacchetti et al. (2021a, 2021b); it is relevant for both credibility and legitimacy since it reduces the ethical responsibility of scientists and protects them from reputational harm.

7 Multi-model ensembles are commonly called “ensembles of opportunity” in that sense.

8 As Cash et al. (2002) have pointed out, there are tensions between salience, credibility and legitimacy (see Section 2).

9 That said, wider uncertainty ranges do not always mean lower credibility. In ensembles of GCMs, the model spread related to climate sensitivity, for example, has not decreased from one IPCC report to another, and yet, this does not mean that GCMs do not provide more credible projections. Quite the opposite, a steady (and even slightly increased) model spread can be explained by GCMs being more and more complex, integrating more and more representations of the climate processes, and thus by the new uncertainty ranges covering previously unmeasured sources of uncertainty (Jebeile and Barberousse 2021).
